# Reducing Peripheral Inflammation with Infliximab Reduces Neuroinflammation and Improves Cognition in Rats with Hepatic Encephalopathy

**DOI:** 10.3389/fnmol.2016.00106

**Published:** 2016-11-02

**Authors:** Sherry Dadsetan, Tiziano Balzano, Jerónimo Forteza, Andrea Cabrera-Pastor, Lucas Taoro-Gonzalez, Vicente Hernandez-Rabaza, Sara Gil-Perotín, Laura Cubas-Núñez, José-Manuel García-Verdugo, Ana Agusti, Marta Llansola, Vicente Felipo

**Affiliations:** ^1^Laboratorio de Neurobiología, Centro Investigación Príncipe FelipeValencia, Spain; ^2^Instituto Valenciano de Patología, Unidad Mixta de Patología Molecular, Centro Investigación Príncipe Felipe/Universidad Católica de ValenciaValencia, Spain; ^3^Unidad Mixta Esclerosis Múltiple y Neurorregeneración, Servicio de Neurología, Fundación Investigación Hospital la FeValencia, Spain; ^4^Laboratorio de Neurobiología Comparada, Institut Cavanilles de Biodiversitat i Biologia-Universidad de ValenciaValencia, Spain; ^5^Instituto de Investigación Sanitaria-INCLIVAValencia, Spain

**Keywords:** hepatic encephalopathy, neuroinflammation, neurotransmission, cognitive impairment, TNF-a

## Abstract

Inflammation contributes to cognitive impairment in patients with hepatic encephalopathy (HE). However, the process by which peripheral inflammation results in cognitive impairment remains unclear. In animal models, neuroinflammation and altered neurotransmission mediate cognitive impairment. Taking into account these data, we hypothesized that in rats with HE: (1) peripheral inflammation is a main contributor to neuroinflammation; (2) neuroinflammation in hippocampus impairs spatial learning by altering AMPA and/or NMDA receptors membrane expression; (3) reducing peripheral inflammation with infliximab (anti-TNF-a) would improve spatial learning; (4) this would be associated with reduced neuroinflammation and normalization of the membrane expression of glutamate receptors. The aims of this work were to assess these hypotheses. We analyzed in rats with portacaval shunt (PCS) and control rats, treated or not with infliximab: (a) peripheral inflammation by measuring prostaglandin E2, IL10, IL-17, and IL-6; (b) neuroinflammation in hippocampus by analyzing microglial activation and the content of TNF-a and IL-1b; (c) AMPA and NMDA receptors membrane expression in hippocampus; and (d) spatial learning in the Radial and Morris water mazes. We assessed the effects of treatment with infliximab on peripheral inflammation, on neuroinflammation and AMPA and NMDA receptors membrane expression in hippocampus and on spatial learning and memory. PCS rats show increased serum prostaglandin E2, IL-17, and IL-6 and reduced IL-10 levels, indicating increased peripheral inflammation. PCS rats also show microglial activation and increased nuclear NF-kB and expression of TNF-a and IL-1b in hippocampus. This was associated with altered AMPA and NMDA receptors membrane expression in hippocampus and impaired spatial learning and memory in the radial and Morris water maze. Treatment with infliximab reduces peripheral inflammation in PCS rats, normalizing prostaglandin E2, IL-17, IL-6, and IL-10 levels in serum. Infliximab also prevents neuroinflammation, reduces microglial activation, translocates NF-kB into nucleoli and normalizes TNF-a and IL-1b content in hippocampus. This was associated with normalization of AMPA receptors membrane expression in hippocampus and of spatial learning and memory. The results suggest that peripheral inflammation contributes to spatial learning impairment in PCS rats. Treatment with anti-TNF-a could be a new therapeutic approach to improve cognitive function in patients with HE.

## Introduction

Hepatic encephalopathy (HE) is a neuropsychiatric syndrome present in patients with liver disease with symptoms ranging from mild cognitive impairment to coma. Around 40% of patients with liver cirrhosis show minimal HE (MHE), with mild cognitive impairment, psychomotor slowing, and attention deficits (Weissenborn et al., 2005; Felipo et al., 2012a) which are not evident but can be unveiled using psychometric tests. MHE affects several million people around the world and impairs their quality of life and the ability to perform daily tasks (Leevy and Phillips, 2007; Bajaj, 2008; Felipo, 2013).

Hyperammonemia and inflammation act synergistically to induce the neurological alterations in MHE and in HE. In cirrhotic patients, hyperammonemia impairs performance in psychometric tests during inflammation but not after its resolution (Shawcross et al., 2004). The serum levels of the pro-inflammatory cytokines IL-6 and IL-18 are higher in cirrhotic patients with MHE than in those without MHE and show a good correlation with the grade of cognitive impairment (Montoliu et al., 2009). The joint presence of certain levels of inflammation and hyperammonemia is enough to induce mild cognitive impairment, even in the absence of liver failure, as shown in a report analyzing neurological impairment in patients with different hepatic or dermatological diseases associated with different grades of inflammation and hyperammonemia (Felipo et al., 2012b).

The mechanisms leading to cognitive impairment in HE seem to involve induction of neuroinflammation which would alter neurotransmission resulting in reduced cognitive function.

Rats with porta-cava shunts (PCS), a main model of HE recommended by the International Society for Hepatic Encephalopathy (Butterworth et al., 2009), show impaired cognitive function and neuroinflammation (Cauli et al., 2007; Agusti et al., 2011). Reducing neuroinflammation with ibuprofen or with inhibitors of MAP kinase p38 improves cognitive function in rats with HE due to PCS (Cauli et al., 2007; Agusti et al., 2011).

An *in vivo* PET study in cirrhotic patients with HE show that they have increased binding in brain of [^11^C](R)-PK11195, a marker of neuroinflammation, correlating with the grade of cognitive impairment (Cagnin et al., 2006). This suggests that patients with HE also show neuroinflammation.

Hyperammonemia *per se* induces neuroinflammation (Rodrigo et al., 2010), but peripheral inflammation may also induce neuroinflammation (Biesmans et al., 2013; Murta et al., 2015). A main aim of this work was to assess whether peripheral inflammation contributes to neuroinflammation and cognitive impairment in rats with HE.

Neuroinflammation would impair cognitive function by altering neurotransmission. Spatial learning and memory are modulated by AMPA and NMDA receptors in hippocampus (Sanderson et al., 2008; Keifer and Zheng, 2010; Wiltgen et al., 2010). Membrane expression of AMPA and NMDA receptors in hippocampus may be altered by neuroinflammation. Exposure to IL-1b reduces membrane expression of GluR1 subunit of AMPA receptors in hippocampal neurons and this seems to be mediated by NMDA receptors (Lai et al., 2006). TNF-a also alters AMPA receptors membrane expression in hippocampus (Ogoshi et al., 2005). These effects of IL-1b and TNF-a would result in altered neurotransmission which would lead to cognitive impairment.

An association between peripheral inflammation and mild cognitive impairment is also present in other diseases leading to chronic inflammation as diabetes, rheumatoid arthritis, obesity or chronic kidney disease (Umemura et al., 2011; Shin et al., 2013; da Matta et al., 2014; Díaz-Gerevini et al., 2014; Nguyen et al., 2014). To reduce peripheral inflammation patients with some of these diseases are being treated with compounds directed to inhibit TNF-a, which plays a pivotal role in the initiation and amplification of the inflammatory cascade (Cheng et al., 2014). In patients with sarcoidosis or rheumatoid arthritis, anti-TNF-a improves cognitive function (Elfferich et al., 2010; Raftery et al., 2012). Anti-TNF-a has been also suggested as a potential treatment against cognitive impairment in Alzheimers disease (Cheng et al., 2014). One anti-TNF-a formulations used in clinical practice is infliximab, a 165 kDa chimeric human-murine monoclonal antibody which binds to both soluble and transmembrane-bound TNF-a forming stable non-dissociating immune complexes. Due to its large size infliximab does not cross the blood-brain-barrier when administered systemically thus specifically targeting peripheral TNF-a (Cheng et al., 2014).

Taking into account the above studies, we hypothesized that in rats with HE:

1. peripheral inflammation would be a main contributor to neuroinflammation;
2. neuroinflammation in hippocampus would impair spatial learning by altering AMPA and/or NMDA receptors membrane expression;
3. reducing peripheral inflammation with infliximab would improve spatial learning;
4. this would be associated with reduced neuroinflammation in hippocampus and normalization of AMPA and/or NMDA receptors membrane expression.

This work aimed to assess these hypotheses. We measured in PCS rats: peripheral inflammation (prostaglandin E2, IL10, IL-17, and IL-6 levels), neuroinflammation in hippocampus (microglial activation and TNF-a and IL-1b content), membrane expression of AMPA and NMDA receptors in hippocampus and spatial learning in the Radial and Morris water mazes.

We assessed the effects of reducing peripheral inflammation by treating PCS rats with infliximab on peripheral inflammation, neuroinflammation in hippocampus, AMPA and NMDA receptors membrane expression and spatial learning and memory.

## Materials and methods

### Portacaval anastomosis and treatment with infliximab

**Figure 1:**
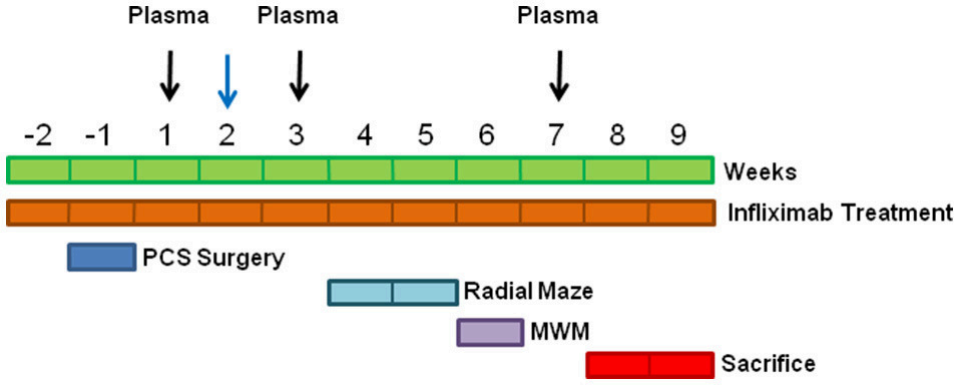
**Scheme showing the experimental design**. Blood was taken at 1, 3 and 7 weeks for ammonia and cytokine determinations (black arrows) and at 1, 2 and 7 weeks to determine PGE2 (blue arrow).

Male Wistar rats (220–240 g) were subjected to portacaval anastomosis as in Lee and Fisher (1961). Control rats were sham operated. The experiments were approved by the Comite de Experimentación y Bienestar Animal of our Center and performed in accordance with guidelines of the Directive of the European Commission (2010/63/EU) for care and management of experimental animals. Animals were distributed into four groups: sham (SM); sham+infliximab (SM INFLIX); PCS; PCS+infliximab (PCS INFLIX). The experiment was repeated four times and eight animals per group were used in each experiment. A total of 32 rats per group were used. Infliximab (Remicade; Merck Sharp &Dohme) was administered i.v. (5 mg/kg) in the tail vein as in Karson et al. (2013). First administration was 2 days before PCS surgery. Weekly treatment with infliximab was maintained until sacrifice except during behavioral tests, when infliximab was administered every 2 weeks. Controls were injected i.v. with saline. The experimental design is summarized in Figure 1.

### PGE2, IL-6, and IL-10 determination in plasma

Blood samples (200 μL) were collected from tail vein at weeks 1, 3, and 7 after PCS surgery and plasma was isolated for determination of ILs. For prostaglandin E2 (PGE2) determination blood (200 μL) was taken and plasma isolated at 1, 2 and 7 weeks after PCS surgery. Prostaglandin E2 was measured using ELISA Biotrak system (Amersham Bioscience, UK). IL-6 and IL-10 levels were analyzed by western blot using primary antibodies against IL-10 (1:1000, Abcam) and IL-6 (1:500, BioSource). Secondary antibodies were anti-rabbit IgG conjugated with alkaline phosphatase (1:4000). The images were captured and band intensities quantified using the AlphaImager 2200 program. Western blot data are given as percentage of controls (sham).

### Ammonia determination in blood

Blood (20 μL) was taken from the tail vein. Blood ammonia was measured immediately after blood collection with the Ammonia Test Kit II for the PocketChemBA system (Arkay, Inc., Kyoto, Japan) following the manufacturer's specifications.

### Brain immunohistochemistry

At week 8 after PCS surgery the rats were anesthetized with sodium pentobarbital and transcardially perfused with 0.9% saline followed by 4% paraformaldehyde in 0.1 M phosphate buffer (pH 7.4). Brains were removed and post-fixed in the same fixative solution for 24 h at 4°C. Five-micrometer thick, paraffin-embedded sections (5 μm) were cut and mounted on coated slide glass. The tissue sections were then processed with the Envision Flex+kit (DAKO) blocking endogenous peroxidase activity for 5 min and then incubated with anti IBA1 (Wako; 1:300 for 30 min), anti TNF-a (Abcam; 1:200 for 45 min), or anti IL-1b (RD SYSTEM; 1:100 for 30 min). The reaction was visualized by incubation with Envision Flex + horseradish peroxidase for 20 min and finally diaminobenzidine for 10 min. Sections were counterstained with Mayer's hematoxylin for 5 min. TNF-a and IL-1b positive cells were manually counted by two blinded experimenters and the results (the mean of two blind experimenters) were expressed as a percentage of the total number of cells. For each rat at least 120–150 cells per section were counted from at least four different sections. Intensity of TNF-a in CA1 region was quantified using ROI manager function in ImageJ (1.48v). CA1 region was selected manually. Inverted values of Mean Gray value were recorded and results expressed as a percentage of control group. For analysis of microglial activation the area of interest was selected. Using Auto Local Threshold and analyzed particle functions in ImageJ, the intensity thresholds and size filter were applied. To measure the perimeter of microglia, the Bernsen method was used and 2000–20,000 size filter was applied. For each rat, at least 30–40 cells were quantified and the results were converted from pixels to micrometers.

### Analysis of TNF-a, IL-1b, IkB, and phosphorylated IkB content by western blot

Rats were sacrificed by decapitation at week 9 after PCS surgery and hippocampi were dissected and homogenized in 66 mM Tris-HCl (pH 7.4), 1% SDS, 1 mM EGTA, 10% glycerol, 1 mM sodium ortho-vanadate, and 1 mM sodium fluoride containing protease inhibitor cocktail (Roche, Mannheim, Germany). Samples were subjected to electrophoresis and immunoblotting as in Felipo et al. (1988) using primary antibodies against TNF-a and IL-1b (RD SYSTEMS; 1:500 for both) and as secondary antibodies anti-goat IgG conjugated with alkaline phosphatase (1:4000) or primary antibodies against IKB alpha (1:5000) and Phospho IKB alpha (S32 + S36) (1:1000) from Abcam and as secondary antibodies anti-rabbit and anti-mouse IgG conjugated with alkaline phosphatase (1:4000). The images were captured and band intensities quantified using the AlphaImager 2200 program. Data are given as percentage of controls (sham).

### Immunofluorescence analysis of NF-κB p65 and p50

Free-floating sections (30 μm) were cut through the hippocampus using vibratome. Parallel series were collected in with 0.1% sodium azide. Sections were washed in 0.1 M phosphate buffer and blocked with normal serum from the same species as the secondary antibody before being incubated overnight with primary antibody (NF-κB p65, 1:200; NF-κB p50, 1:200; Fibrillarin, 1:300) from Abcam, diluted in blocking buffer and secondary fluorescent antibody (1:400) from Invitrogen. The nuclei were stained with DAPI (Sigma-Aldrich) and sections were mounted on slides and cover-slipped. A negative control was performed omitting the primary antibodies. The images were observed under confocal microscope (Leica TCS-SP2-AOBS) and photographically recorded.

p50 and p65 may be located in the nuclei or in the cytosol. The nuclei were labeled in blue with DAPI. Nuclear NFKB subunits (p50 or p65) were quantified as the green puncta inside blue staining (DAPI). p65 or p50 outside the blue staining are in the cytosol. Green puncta outside DAPI staining was quantified as cytosolic NFKB. Nuclear intensity of both NFKB subunits was analyzed using ImageJ (1.48v). Nuclei were outlined using ROI manager function on DAPI blue channel and the selection was applied on green channel (p50 or p65 channel) to measure nuclear fluorescence. Mean Gray value for each nucleus was measured. At least 120 cells per section were counted from at least eight different sections. NFkB marked nucleoli were manually counted by two blinded experimenters. The ratio of nucleoli/cells (the mean of the data obtained by two blinded experimenters) was calculated and expressed as a percentage respect to control. A double immunofluorescence was performed, using the nucleoli marker fibrillarin (Abcam, 1:300) and p50 subunit of NF-κB to confirm nucleolar traslocation.

### Fluorescence *In situ* hybridization

Fluorescence *in situ* hybridization was performed to detect TNF-a mRNA expression in 5 μm hippocampal sections. Slides were deparaffined and rehydrated. Tissue was digested with 5 μg/ml proteinase K (Ambion-Life Technologies) in DEPC water for 6 min at room temperature. A fluorescein-conjugated probe of 23 nucleotides (50 μM; Exiqon) was diluted in hybridization solution (50 ng/μl) with 30% formamide and denatured at 80°C for 2 min. It was immediately chilled on ice to prevent re-annealing. The slices were incubated for 16 h in a humidified hybridization chamber at 60°C. The next day 2 stringency washes were performed with 1X SSC at 48°C for 15 min and 1X SSC at room temperature for 15 min. The slices were counterstained with 4′,6-diamidino-2-phenylindole (DAPI; Sigma; 5 μg/ml) and Neun (Millipore, 1:100). The results were observed under confocal microscope and photographically recorded.

### Membrane expression of receptors

At week 8 after PCS surgery rats were sacrificed and brains rapidly removed and dropped into ice-cold standard buffer (in mM): NaCl 121, KCl 1.87, KH_2_PO_4_ 1.17, NaHCO_3_ 26.2, CaCl_2_ 2.5, and glucose 11, aerated with 95% O_2_-5% CO_2_ (pH 7.4). Hippocampi were dissected and transversal slices (400 μm) added to tubes containing ice-cold standard buffer with or without 2 mM BS3 (Pierce, Rockford, IL). Samples treated or not with BS_3_ were analyzed by Western blot as in Boudreau and Wolf (2005). Primary antibodies used were: anti-GluR1 (1:500), anti-GluR2 (1:2000), and anti-NR2A (1:1000) from Millipore (Temecula, CA, USA) and anti-NR1 (1:1000) from BD Bioscience (Franklin Lakes, NJ, USA). Treatment with BS3 aggregates all proteins present in the cell membrane leaving intact intracellular proteins. In the western blot, in the samples obtained in the absence of BS3 the band stained by the antibody contains all the antigen (e.g., GluR1) present in the sample, both in membrane and intracellular. In the samples treated with BS3 the membrane proteins are aggregated and do not enter the gel. So that the band stained contains only the intracellular (non-membrane) antigen. The membrane expression of the receptors was calculated as the difference between the intensity of the bands without BS3 (total protein) and with BS3 (non-membrane protein).

### Spatial learning in 8 arms radial maze

The rats had to locate food pellets placed at the end of 4 out of 8 arms according to a random configuration as in Hernandez-Rabaza et al. (2010). Each animal performed three trials per day during 3 days. The number of spatial reference errors (entry to unbaited arms) and working memory errors (number of entries to arms already visited in the same trial) were recorded.

### Spatial learning and memory in the morris water maze

Spatial learning and memory in the Morris water Maze was analyzed as in Monfort et al. (2007) 6 weeks after PCS surgery. Rats were trained to learn the fixed location of the invisible platform during 4 days with 3 swims per day. Seventy-two hours after last training day the platform was removed and the rats were allowed to swim for 90 s. The time spent in the quadrant in which was the platform was recorded.

### Statistical analysis

**Figure 2:**
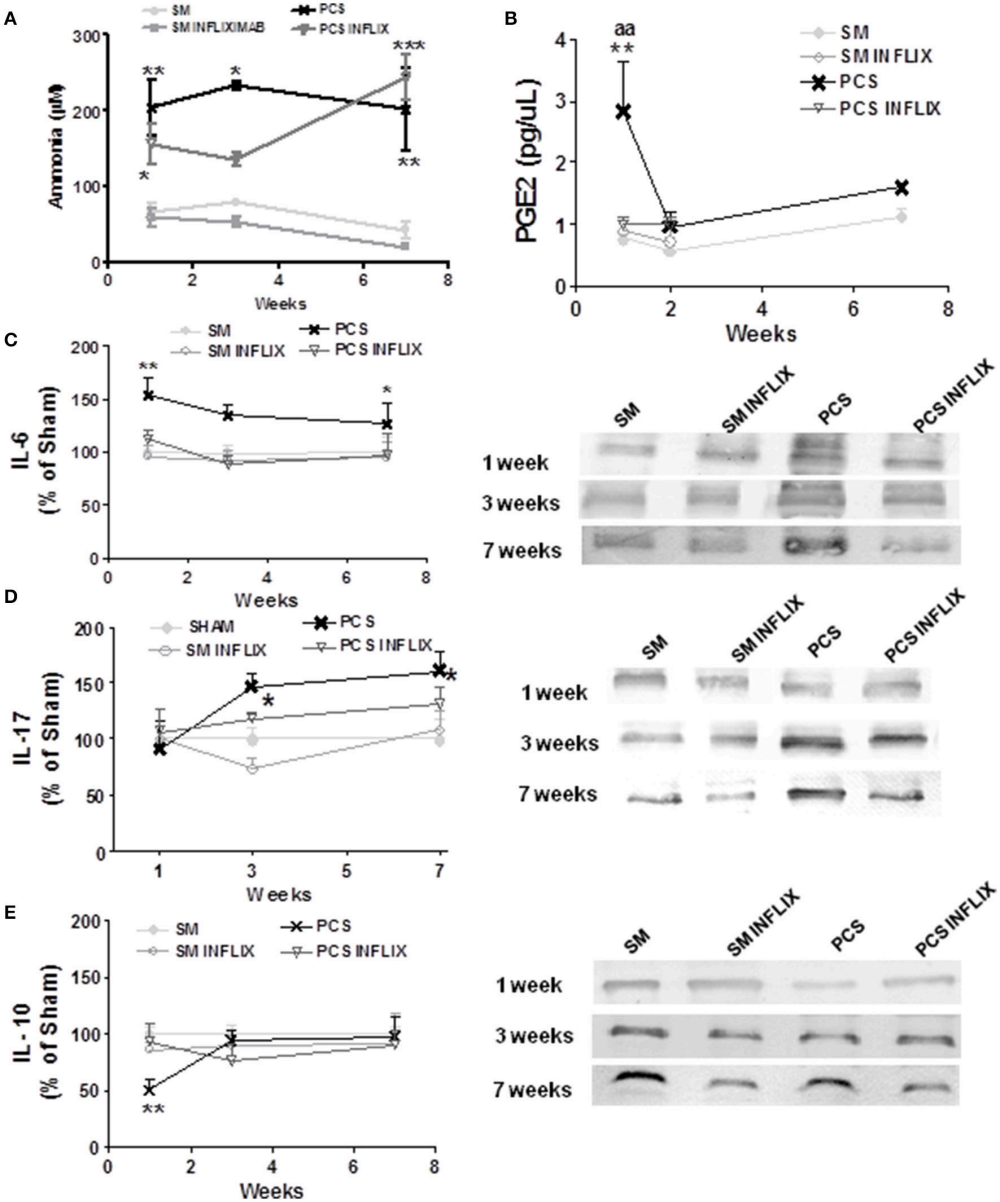
**Infliximab reduces peripheral inflammation but not hyperammonemia in PCS rats**. Blood samples were taken at the indicated times after surgery from control (sham, SM) or PCS rats treated with vehicle or infliximab (INFLIX). **(A)** Ammonia levels were measured in blood. Plasma was isolated from blood samples and PGE2 levels **(B)**, IL-6 **(C)**, IL-17 **(D)**, and IL-10 **(E)** were analyzed. Representative images of western blots are shown. Values are mean ± SEM of 5 rats per group for PGE2 and 8–14 rats per group for IL-6 and IL-10. Two-way ANOVA with repeated measures and Bonferroni post-test were performed. Statistic values for ammonia **(A)** were: *F* = 23.3, Df = 3, *P* < 0.0001. For PGE2 **(B)** the effects of PCS and Infliximab treatment were statistically different with *p* < 0.05, *F* = 7.3 and Df = 3 and there is also a significant effect of time (*p* < 0.05, *F* = 5.8, Df = 1). For IL-6 **(C)** PCS rats were statistically different from controls with *p* < 0.01, *F* = 13 and Df = 1 and the effect of Infliximab treatment in PCS rats was also statistically significant (*p* < 0.01, *F* = 9.4, Df = 1). For IL-17 **(D)** PCS rats were statistically different from controls with *p* < 0.05, *F* = 3.8 and Df = 3 whereas the effect of Infliximab treatment in PCS rats was not statistically significant. No time effect was found in this case. For IL-10 **(E)** values for PCS rats were statistically different from controls with *p* < 0.05, *F* = 6.4 and Df = 1 and there is also a significant effect of time (*p* < 0.05, *F* = 4.0, Df = 2). Interaction was significantly different (*p* < 0.05, *F* = 4.0, *f* = 2). Values significantly different from controls are indicated by asterisks and from PCS rats by aa. ^*^p ≤ 0.05; ^**^p < 0.01; ^***^p < 0.001; ^aa^*p* < 0.01.

Results are expressed as mean ± SEM. Values are given in international units when possible (ammonia, PGE-2). Values which are relative (immunohistochemistry, immunofluorescenc, Western blot,) are expressed as percentage of control rats. Data were analyzed by one-way ANOVA followed by Tukey *post-hoc* test, except for Figures 2A,C–E and 9A,C,E, where two-way ANOVA with repeated measures was done. *P* < 0.05 is considered significant differences.

## Results

### Infliximab reduces peripheral inflammation but not ammonia levels in PCS rats

PCS rats show increased (*p* < 0.0001) ammonia levels, ranging between 200 and 240 μM at 1, 3, and 7 weeks while in control (sham) rats ammonia ranges between 40 and 80 μM (Figure 2A). Treatment with infliximab did not affect ammonia levels in sham or PCS rats. Ammonia levels in PCS rats treated with infliximab ranged between 180 and 245 μM at 1, 3, and 7 weeks (Figure 2A).

PCS rats show peripheral inflammation with a rapid increase of prostaglandin 2 (PGE2) levels which reach 369 ± 98% (*p* < 0.01) of controls 1 week after surgery. Treatment with infliximab prevents PGE2 increase, keeping it at 131 ± 13% of controls. PGE2 in PCS rats return to normal levels (150 ± 25% of controls) 2 weeks after surgery, remaining at this level at 7 weeks (142 ± 11% of controls) (Figure 2B).

Pro-inflammatory IL-6 in serum of PCS rats increase to 147 ± 12% (*p* < 0.01), 123 ± 7%, and 135 ± 6% (*p* < 0.05) of controls at 1, 3, and 7 weeks, respectively (Figure 2C).

Treatment with infliximab keeps IL-6 at normal values at all times tested, reaching 113 ± 10, 89 ± 7, and 109 ± 14% of controls at 1, 3, and 7 weeks, respectively (Figure 2C).

Pro-inflammatory IL-17 in serum of PCS rats increase to 146 ± 12 and 162 ± 16% (*p* < 0.05) of controls at 3 and 7 weeks, respectively (Figure 2D).

Treatment with infliximab keeps IL-17 at lower values, reaching 118 ± 6 and 132 ± 15% of controls at 3 and 7 weeks, respectively, not statistically different from control rats (Figure 2D).

Peripheral inflammation in PCS rats is also reflected in anti-inflammatory IL-10, which decreases to 56 ± 9 and 74 ± 11% (*p* = 0.02) of controls at 1 and 3 weeks, respectively. At 7 weeks IL-10 returned to normal levels (99 ± 13%) in PCS rats (Figure 2E).

Infliximab prevents IL-10 decrease in PCS rats, especially at 1 week. IL-10 remained at 80 ± 12 and 77 ± 15% of controls at 1 and 3 weeks, respectively (Figure 2E).

Infliximab reduces therefore peripheral inflammation in PCS rats.

### PCS rats show neuroinflammation in hippocampus which is reduced by infliximab

**Figure 3:**
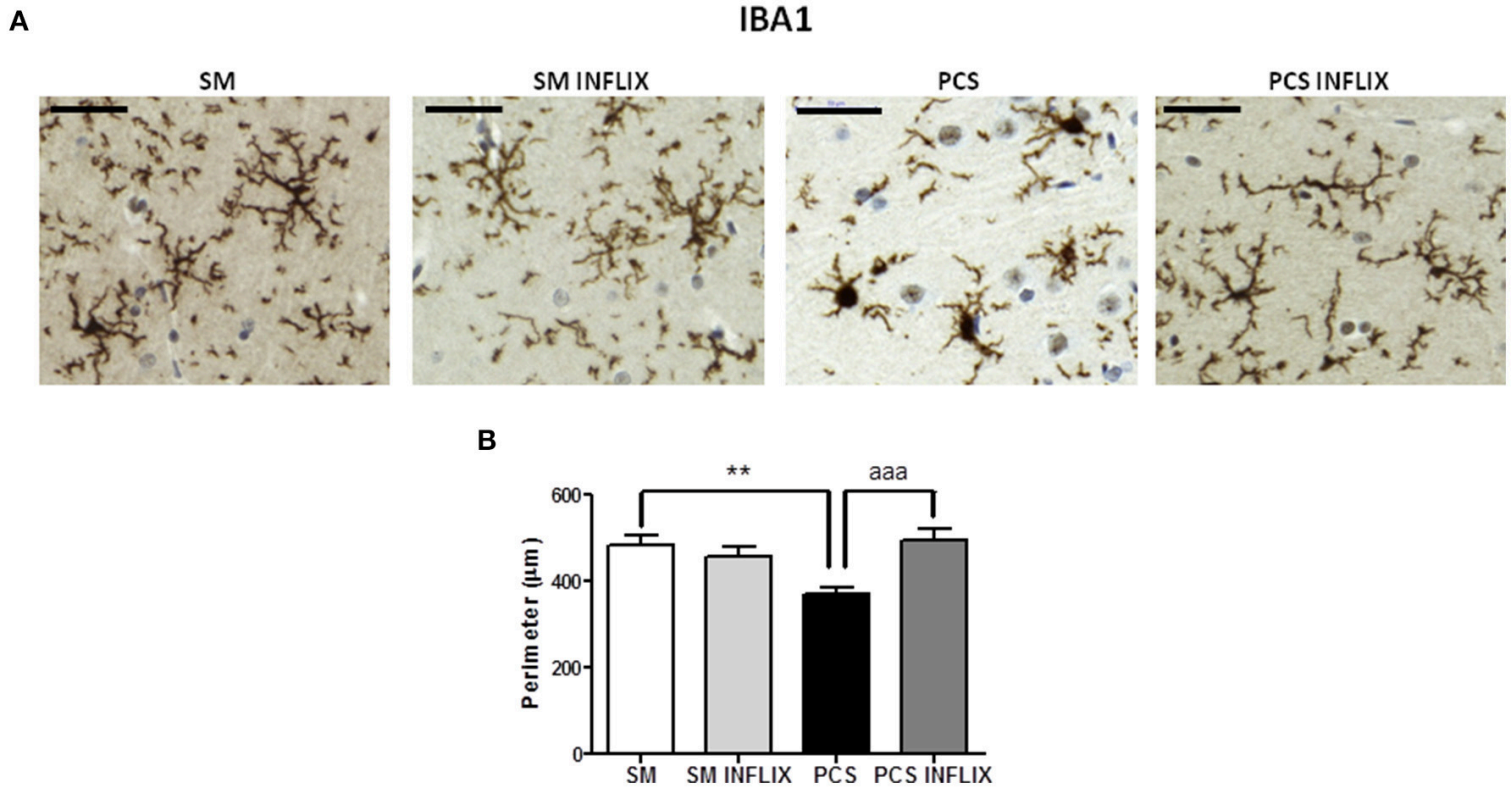
**Infliximab reduces microglial activation in the hippocampus of PCS rats**. Rats were sacrificed and hippocampus dissected 8 weeks after PCS surgery. Immunohistochemistry was performed as indicated in the “Materials and Methods” section using antibody against Iba-1. Representative images are shown **(A)**. The perimeter of microglial cells was quantified **(B)**. One-way ANOVA with Tukey *post-hoc* test was performed to compare all groups. Differences between groups were statistically different: *F* = 7.5, Dfn = 3, Dfr = 198, *p* < 0.0001; and variances were not statistically different. Values are the mean ± SEM of 4 rats per group. Values significantly different from controls are indicated by asterisks and from PCS rats by a. ^**^p < 0.01; ^aaa^*p* < 0.005. Scale bar = 50 μm.

PCS rats show activation of microglia in hippocampus, with increased cell body size and shorter processes compared to control rats (Figure 3A). As a measure of the grade of activation we analyzed the perimeter of microglial cells stained with Iba1 (Figure 3B): The perimeter was lower (*p* < 0.001) in PCS rats (368 ± 19 μm) than in control rats (481 ± 25 μm). In PCS rats treated with infliximab the perimeter of microglial cells was 495 ± 27 μm, which is not different from controls, indicating prevention of microglial activation following infliximab treatment of PCS rats.

**Figure 4:**
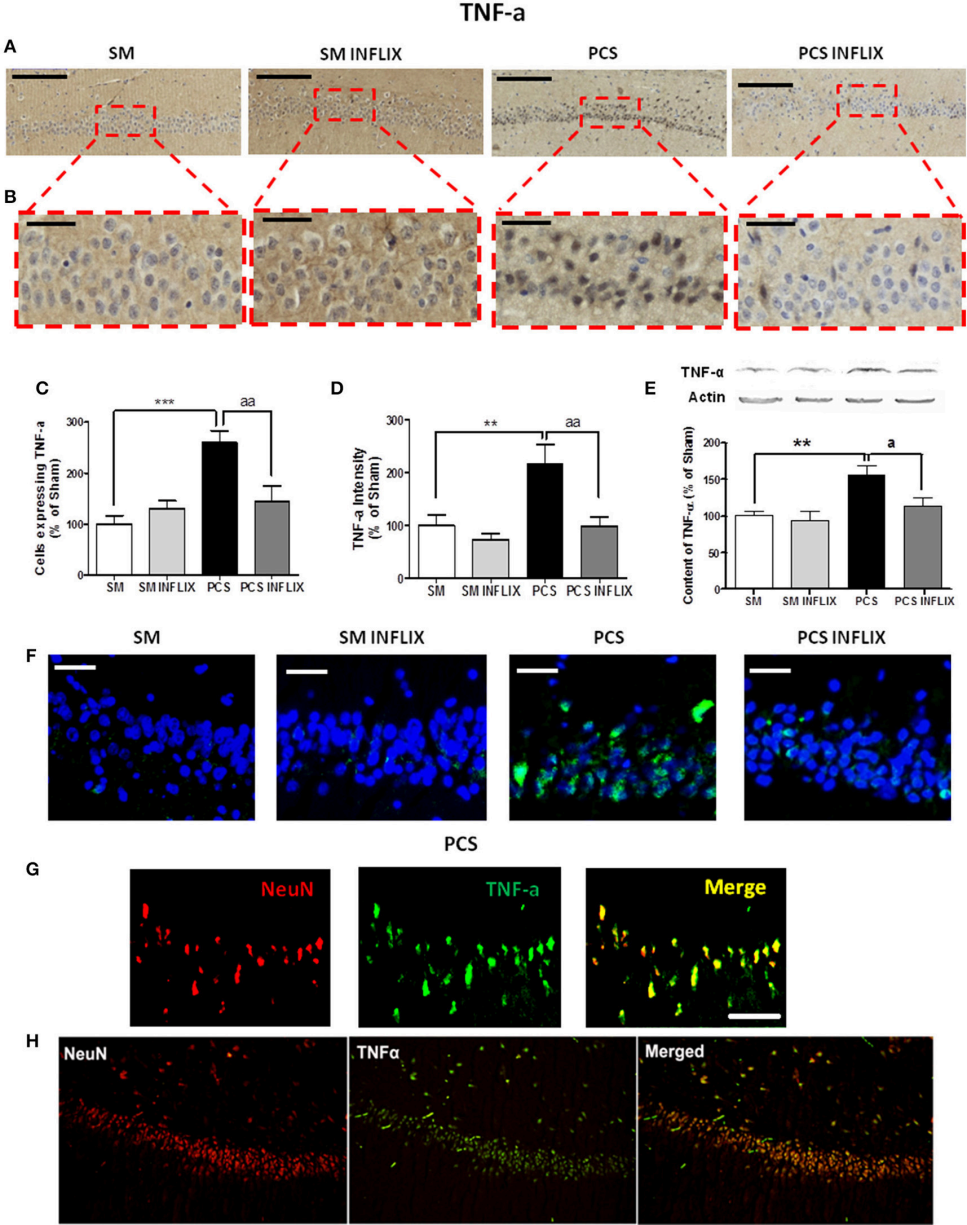
**TNF-a levels are increased in hippocampus of PCS rats and are normalized by infliximab**. Rats were sacrificed and hippocampus dissected 8 weeks after PCS surgery. Immunohistochemistry was performed using anti-TNF-a. **(A)** Representative low (scale bar = 200 μm) and **(B)** high (scale bar = 50 μm) magnification images are shown. The number of cells expressing TNF-a **(C)** and intensity of the staining for TNF-a **(D)**, were quantified. One-way ANOVA with Tukey *post-hoc* test was performed to compare all groups. Differences between groups were statistically different: **(B)** *F* = 9.4, Dfn = 3, Dfr = 52, *p* < 0.0001; **(C)** *F* = 7.3, Dfn = 3, Dfr = 51, *p* < 0.001; and variances were not statistically different. Values are the mean ± SEM of 4 rats per group. For each rat at least 120–150 cells from four different sections were counted. **(E)** Western blot was performed as described in method. Values are the mean ± SEM of 10–14 rats per group. One-way ANOVA with Tukey's *post-hoc* test was performed to compare all groups. The differences between groups were statistically different (*p* < 0.001, *F* = 6.9, Df between groups = 3) and variance was not statistically different. **(F)** Representative images of *in situ* hybridization for TNF-a mRNA (green color; scale bar = 50 μm). **(G)** Double fluorescence staining of the neuronal marker NeuN (red), TNF-a mRNA (green), and merged (yellow) showing co-localization in PCS rats (Scale bar = 50 um). **(H)** Low magnification images (10 ×) of the neuronal marker NeuN (red), TNF-a mRNA (green), and merged (yellow) showing co-localization in PCS rats. Values significantly different from controls are indicated by asterisks and values different from PCS rats by a. ^a^*p* < 0.05; ^**^p < 0.01; ^***^p ≤ 0.005; ^aa^*p* < 0.01.

Figures 4A,B show representative low and high magnification images of immunohistochemistry analysis for TNF-a in the CA1 region of hippocampus. PCS rats show a strong increase (260 ± 23% of controls, *p* < 0.001) in the number of cells expressing TNF-a. Treatment with infliximab reduced it to normal levels (145 ± 31% of controls) (Figure 4C). Moreover, the staining intensity with anti-TNF-a increased in PCS rats to 216 ± 38% of controls (*p* < 0.001) but not in PCS rats treated with infliximab (98 ± 18% of controls) (Figure 4D). Content of TNF-a in hippocampus was also analyzed by western blot. PCS rats show increase of TNF-a to 155 ± 13% of controls (*p* < 0.01) but not PCS rats treated with infliximab (114 ± 11% of controls, *p* < 0.05 compared with PCS rats) (Figure 4E).

The staining for TNF-a is mainly observed in neurons. To assess whether this is due to TNF-a synthesis into the neurons we used fluorescence *in situ* hybridization to visualize TNF-a mRNA. PCS rats show a high expression of TNF-a mRNA (in green) in neurons of the CA1 region which is much lower in PCS rats treated with infliximab. TNF-a mRNA was practically absent in neurons of control rats (Figure 4F).

To further confirm that the mRNA for TNF-a in PCS rats is located in neurons we assessed, by double immunofluorescence labeling, if it co-localizes with the neuronal marker NeuN. As shown in Figures 4G,H, this is the case, confirming neuronal expression of TNF-a in PCS rats.

**Figure 5:**
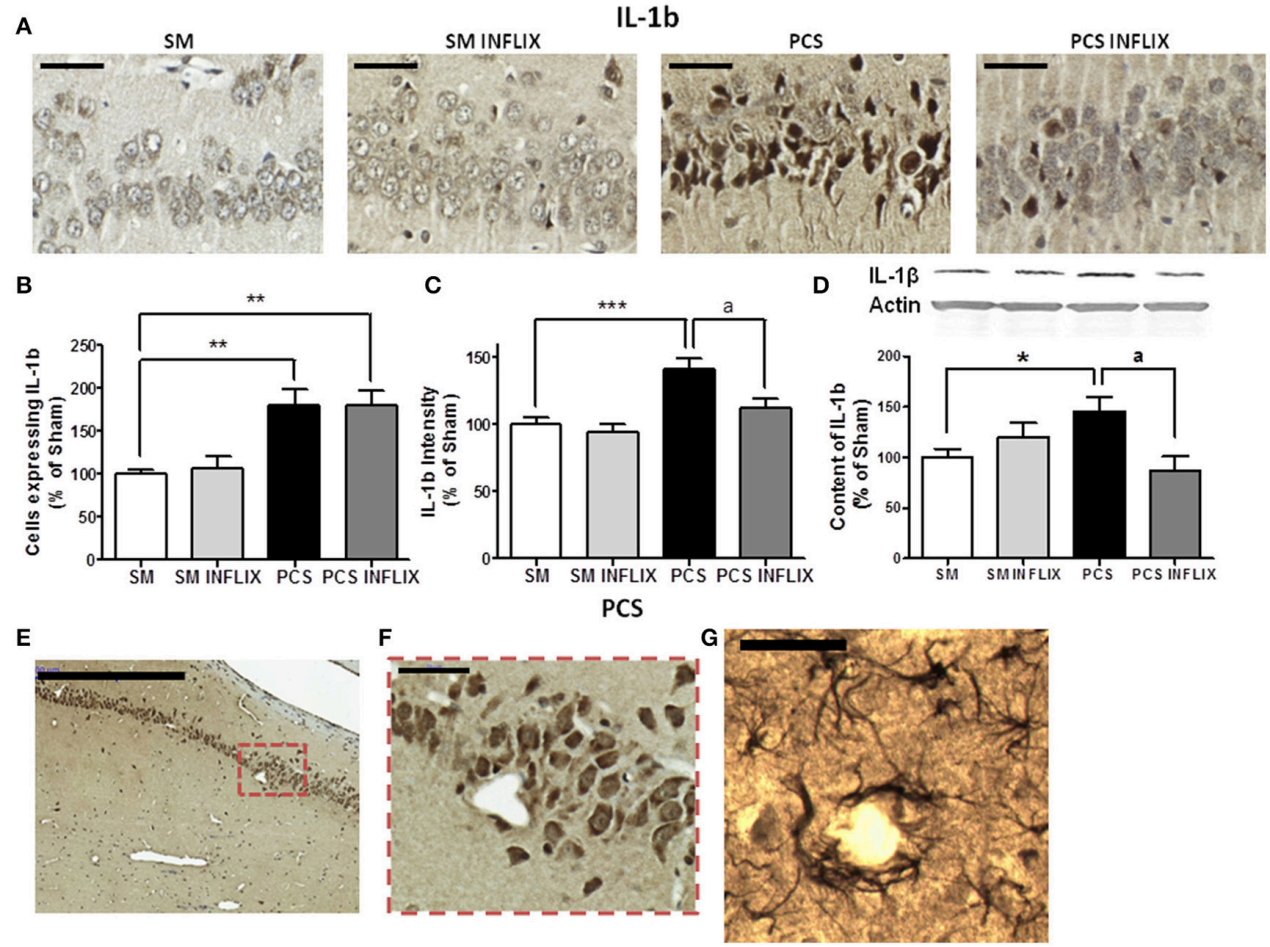
**IL-1b levels are increased in hippocampus of PCS rats and are normalized by infliximab**. Rats were sacrificed and hippocampus dissected 8 weeks after PCS surgery. Immunohistochemistry was performed using antibodies against IL-1b (**A**; scale bar = 50 μm). The number of cells expressing IL-1b **(B)** and staining intensity **(C)** for IL-1b were quantified. One-way ANOVA with Tukey *post-hoc* test was performed to compare all groups. Differences between groups were statistically different: **(B)** *F* = 8.1, Dfn = 3, Dfr = 49, *p* < 0.001; **(C)** *F* = 9.1, Dfn = 3, Dfr = 46, *p* < 0.0001 and variances were not statistically different. Values are the mean ± SEM of 4 rats per group in **(B)** and six rats per group in **(C)**. For each rat at least 120–150 cells from four different sections were counted. **(E,F)** Representative low (scale bar = 500 μm) and high (scale bar = 50 μm) magnification images showing neurons expressing IL-1b around a blood vessel. **(G)** Representative image showing astrocytes stained with the IL-1b antibody around a blood vessel (scale bar = 50 μm). Western blot was performed using anti-IL-1b **(D)** as described in methods. Values are the mean ± SEM of 8–10 rats per group. One-way ANOVA with Tukey's *post-hoc* test was performed to compare all groups. The differences between groups were statistically different (*p* < 0.05, *F* = 3.2, Df between groups = 3) and the variance was not statistically different. Values significantly different from controls are indicated by asterisks and values different from PCS rats by a. ^*^p < 0.05; ^**^p < 0.01; ^***^p ≤ 0.005; ^a^*p* < 0.05.

For IL-1b, a representative image of the immunohistochemistry analysis in the CA1 region of hippocampus is shown in Figure 5A. The number of cells expressing IL-1b was increased in PCS rats to 180 ± 19% of controls (*p* < 0.001) and was not affected by infliximab (180 ± 18%) (Figure 5B). The staining intensity for IL-1b increased in PCS rats (142 ± 8%, *p* < 0.001) compared to controls, indicating increased IL-1b levels. Treatment with infliximab normalized IL-1b levels to 112 ± 7% of controls (Figure 5C).

To confirm the effects of PCS and infliximab on the content of TNF-a and IL-1b in hippocampus, we quantified them by Western blot. The content of TNF-a was increased (*p* < 0.05) in PCS rats to 144 ± 17% of controls and was normalized by infliximab (119 ± 11% of controls) (Figure 4D). The content of IL-1b was increased (*p* < 0.05) in PCS rats to 138 ± 15% of controls, and was normalized by infliximab (87 ± 14% of controls) (Figure 5D). We observed that many cells labeled with IL-1b are located surrounding blood vessels, as illustrated in Figures 5E–G.

**Figure 6:**
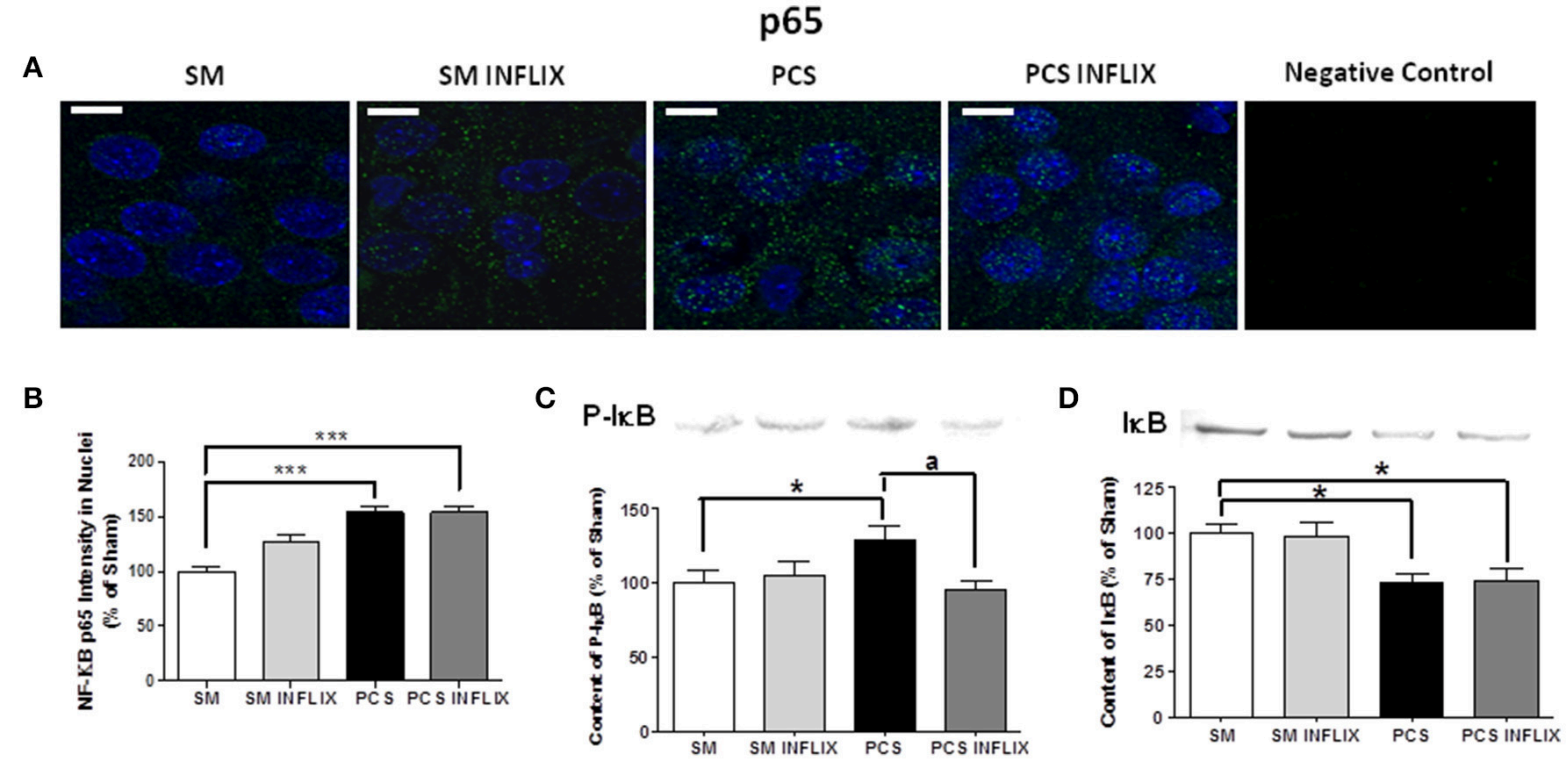
**Effects of infliximab on p65 subunit of NF-kB content in nuclei and on IkB**. Rats were sacrificed and hippocampus dissected 8 weeks after PCS surgery. **(A)** Immunofluorescence was performed using antibodies against the p65 subunit of NF-kB (green staining). Nuclei were stained in blue with DAPI. **(B)** The intensity of p65 staining in nuclei was quantified. Values are mean ± SEM of 3 rats per group. For each rat at least 120 cells per section were counted in at least eight different sections. One-way ANOVA with Tukey's *pos-hoc* test was performed: statistic values were *F* = 25.5, Dfn = 3, Dfr = 511 and *p* < 0.0001. **(C)** Content of IkB phosphorylated in S132 + S36 was analyzed by western blot. Values are mean ± SEM of 3 rats per group. One-way ANOVA with Tukey's *pos-hoc* test was performed: statistic values were *F* = 2.5, Dfn = 3, Dfr = 24 and *p* < 0.05. **(D)** Content of IkB was analyzed by western blot. Values are mean ± SEM of 3 rats per group. One-way ANOVA with Tukey's *pos-hoc* test was performed: statistic values were *F* = 5.6, Dfn = 3, Dfr = 28 and *p* < 0.01. Values significantly different from controls are indicated by asterisks. ^*^p < 0.05, ^***^p ≤ 0.005. Values significantly different from PCS rats are indicated by ^a^*p* < 0.05.

**Figure 7:**
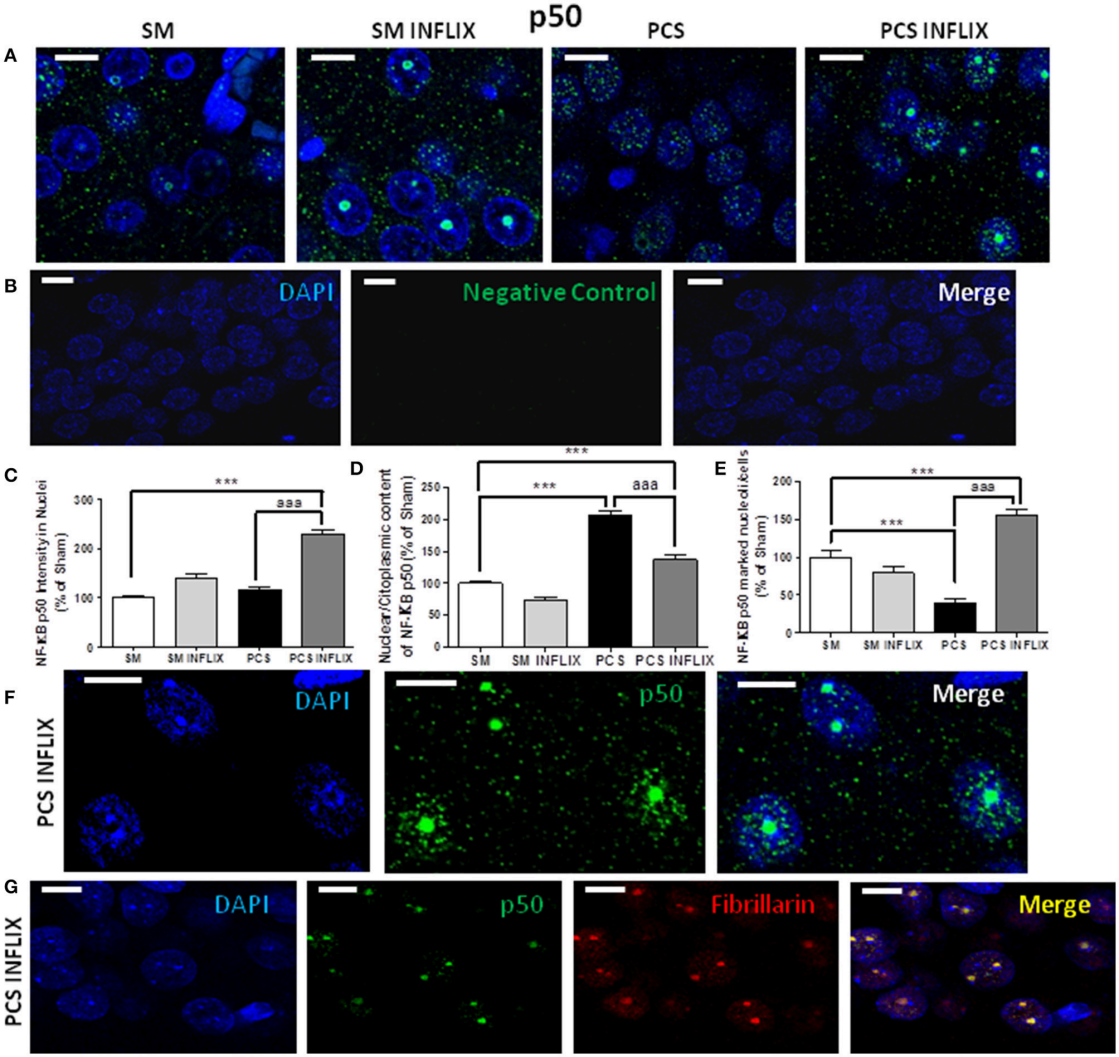
**Effects of infliximab on p50 subunit of NF-kB content in nuclei and nucleoli**. Rats were sacrificed and hippocampus dissected 8 weeks after PCS surgery. **(A)** Immunofluorescence was performed using antibodies against the p50 subunit of NF-kB (green staining). Nuclei were stained in blue with DAPI. A negative control performed in the absence of p50 subunit antibody is shown in **(B)**. Double immunofluorescence was performed using NF-kB p50 (green) and Fibrillarin, a marker of nucleoli (red). The merge show co-localization (yellow) of these proteins **(G)**. Representative images and split channels of NF-kB p50 immunofluorescence **(F)** are shown. The intensity of p50 **(C)** staining in nuclei, the ratio of p50 in nuclei/cytoplasm **(D)** and the proportion of cells containing p50 into the nucleoli **(E)** were quantified. Values are mean ± SEM of 3 rats per group. For each rat at least 120 cells per section were counted in at least eight different sections. One-way ANOVA with Tukey's *pos-hoc* test was performed: in **(C)** statistic values were *F* = 50.6, Dfn = 3, Dfr = 960, *p* < 0.0001; in **(D)**, *F* = 54, Dfn = 3, Dfr = 81, *p* < 0.0001; in **(E)**, *F* = 97.8, Dfn = 3, Dfr = 810, *p* < 0.0001; Values significantly different from controls are indicated by asterisks and values different from PCS rats by aaa. ^***^p ≤ 0.005; ^aaa^*p* < 0.005. Scale bar = 10 μm.

As expression of TNF-a and IL-1b is modulated by the transcription factor NF-kB, we assessed whether infliximab could be normalizing TNF-a and IL-1b expression acting through NF-kB. Representative immunostaining images of p65 and p50 and subunits of NF-kB are shown in Figures 6A, 7A. It can be seen that anti-p50 stained the nucleoli within the nuclei (Figures 7A,B), while p65 did not (Figure 6A).

The intensity of the p65 subunit of NF-kB staining in the nuclei was increased in PCS rats to 154 ± 6% of controls. Treatment with infliximab did not affect this increase, which remained at 154 ± 6% of controls (Figure 6B).

The increased translocation of p65 to the nuclei in PCS rats was associated with increased phosphorylation of IkB, which reached 130 ± 9% (*p* < 0.05) of control rats (Figure 6C) and degradation of IkB, which levels were reduced to 73 ± 5% (*p* < 0.05) of control rats (Figure 6D). Treatment with infliximab reduced the phosphorylation of IkB to 95 ± 6% of control rats (Figure 6C) and did not affect degradation of IkB, which levels remained at 75 ± 6% (*p* < 0.05) of control rats (Figure 6D). The lack of effect of infliximab on IkB content is in agreement with the lack of effect on nuclear p65 shown above.

More remarkable effects were found on the p50 subunit of NF-kB. In PCS rats the intensity of the p50 subunit of NF-kB in the nuclei was not affected. Treatment with infliximab increased it in PCS rats to 228 ± 10% of controls (Figure 7C).

The main effects were found in the subcellular localization of p50 between cytosol-nuclei and, especially, in nucleoli. The ratio of p50 in the nuclei (green puncta inside DAPI staining) vs. cytosol (green puncta outside DAPI staining) was increased in PCS rats to 207 ± 7% of controls (*p* < 0.001). Treatment with infliximab reduced this ratio to 137 ± 6% of controls (Figure 7D).

In PCS rats the number of the cells expressing p50 in the nucleoli was strongly reduced (40 ± 5%; *p* < 0.001) (Figure 7E). Treatment with infliximab induced a strong increase (to 156 ± 8% of control rats) in the number of cells expressing p50 in the nucleoli. Infliximab did not affect p50 in nucleoli in control rats, which remained at 79 ± 8% of the cells (Figure 7E). Treatment of PCS rats with infliximab induces a massive translocation of p50 from the nuclei inside the nucleoli (Figure 7F).

To confirm that infliximab induces a translocation of p50 to nucleoli we performed a double immunofluorescence labeling of a marker of nucleoli (fibrillarin, in red) and of p50 (green). The images in Figure 7G show that there is a co-localization of p50 and fibrillarin, thus confirming the translocation of p50 to the nuclei in PCS rats treated with infliximab.

### Membrane expression of AMPA and NMDA receptors is altered in hippocampus of PCS rats but not in PCS rats treated with infliximab

**Figure 8:**
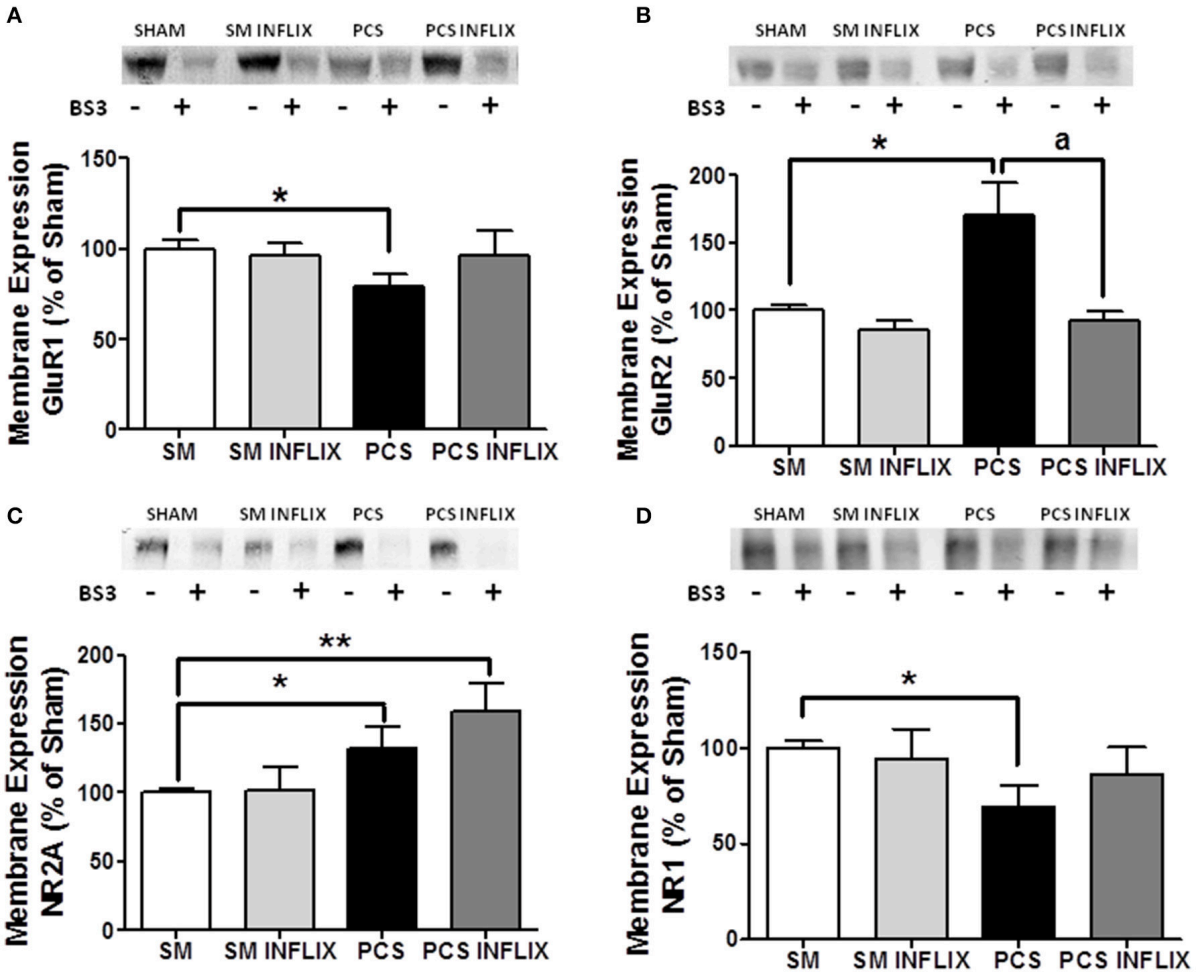
**The membrane expression of the GluR1 and GluR2 subunits of AMPA receptors and NR1 and NR2A subunits of NMDA receptors is altered in hippocampus of PCS rats and is normalized by treatment with infliximab**. Rats were sacrificed and hippocampus dissected 8 weeks after PCS surgery. Samples incubated in the absence (−) or presence (+) of BS3 were subjected to Western blotting using antibodies for each subunit. Representative images are shown. Samples without BS3 represent the total amount of each protein. Samples with BS3 correspond to the non-membrane fraction. The band intensities were quantified and membrane expression was calculated as the difference of intensity between samples without and with BS3. Values are expressed as percentage of controls and are mean ± standard errors of 8–16 rats per group. One-way ANOVA with Tukey's *post-hoc* test was performed to compare all groups. For **(A)** GluR1 differences between groups were statistically different (*p* < 0.05, *F* = 1.5, Df between groups = 3). For **(B)** GluR2 statistics was *p* < 0.05, *F* = 3.2, Df between groups = 3. For **(C)** NR2A statistics was *p* < 0.001, *F* = 10.0, Df between groups = 3. For **(D)** NR1 statistics was *p* < 0.001, *F* = 1.3, Df between groups = 3. In all cases variances were not statistically different. Values significantly different from controls are indicated by asterisks and values different from PCS rats by a. ^*^p < 0.05; ^a^*p* < 0.05; ^**^p < 0.01.

The expression in membrane of the GluR1 subunit of AMPA receptors was reduced (*p* < 0.05) in hippocampus of PCS rats to 79 ± 9% of control rats (Figure 8A). In contrast, membrane expression of the GluR2 subunit was increased (*p* < 0.05) in PCS rats to 150 ± 30% of control rats (Figure 8B).

Infliximab normalized the membrane expression of both GluR1, to 93 ± 13% of control rats (Figure 8A) and GluR2, to 108 ± 8% of control rats (Figure 8B). These values are not different from control rats, indicating prevention of the effect of PCS.

Concerning NMDA receptors, the membrane expression of the NR2A subunit is increased (*p* < 0.05) in PCS rats to 132 ± 16% of control rats (Figure 8C). In contrast, membrane expression of the NR1 subunit was reduced (*p* < 0.05) in PCS rats to 75 ± 11% of control rats (Figure 8D).

Infliximab normalized the membrane expression of NR1, to 108 ± 20% of control rats (Figure 8D) but not that of NR2A, that reached 158 ± 20% of control rats (Figure 8C).

### Spatial learning and memory are impaired in PCS rats but not in PCS rats treated with infliximab

**Figure 9:**
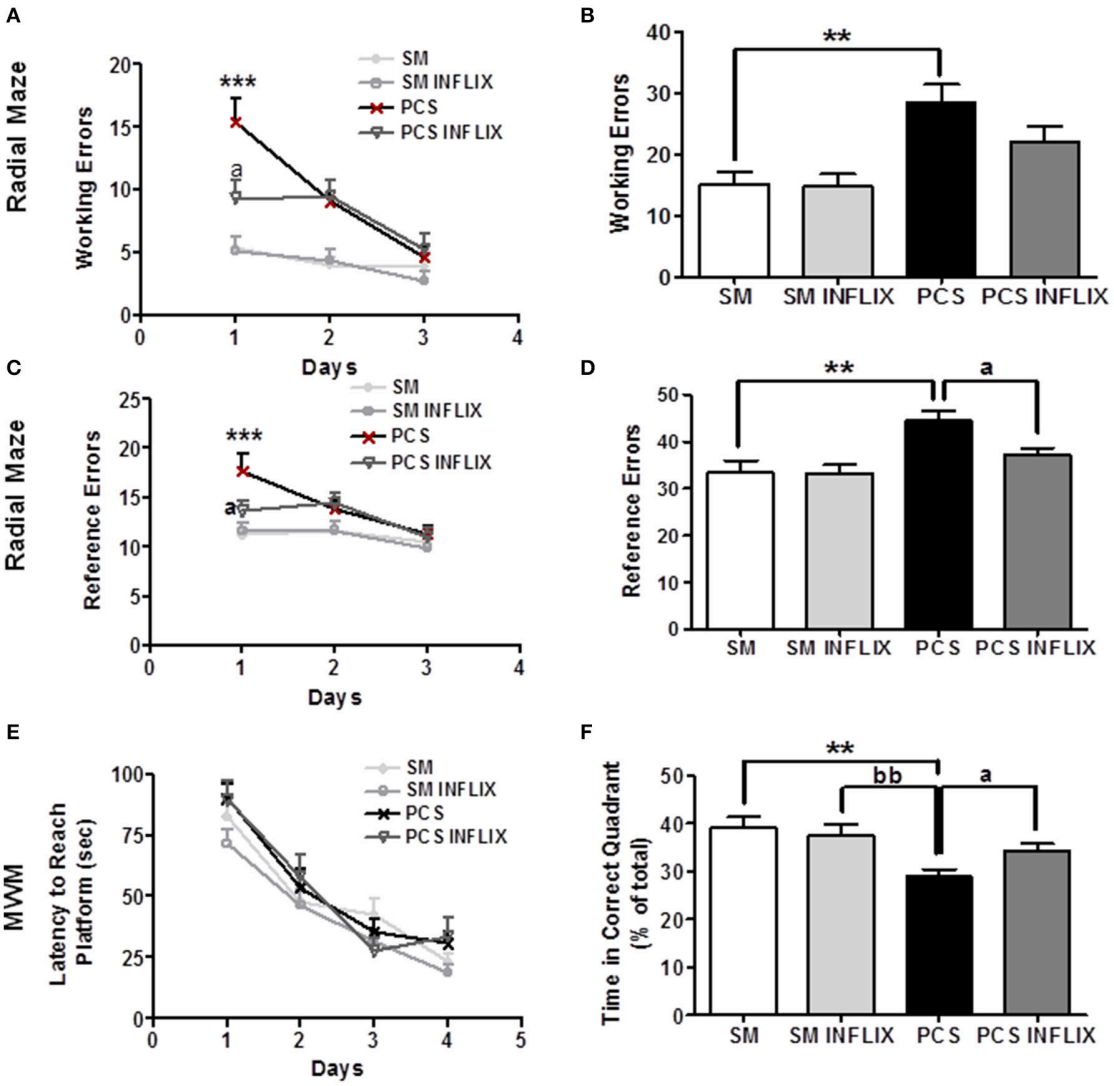
**Spatial learning and memory in the radial and Morris water mazes are impaired in PCS rats and restored by infliximab**. Radial maze was performed from 4 to 5 weeks and MWM at 6 weeks after PCS surgery. Spatial learning in the radial maze was assessed in sham (SM) and PCS rats, treated with vehicle or infliximab (INFLIX). Working **(A,B)** and reference **(C,D)** errors were counted on each day **(A,C)** and in the total period of 3 days **(B,D)**. Spatial learning and memory was also assessed in the Morris water maze. Escape latencies (in seconds) to reach the platform during the different sessions are shown in **(E)** and the time spent in the right quadrant in the memory test in **(F)**. Values are mean ± SEM of 15–16 rats per group. Two-way ANOVA with repeated measures and Bonferroni post-test were performed in **(A,C,E)**. Statistic values were: *F* = 10, Df = 3, *p* < 0.0001 comparing all groups; *F* = 17.5, Df = 2, *p* < 0.0001 for the effect of time and for interaction, *F* = 4.3, Df = 6, *p* < 0.001 in **(A)**. In **(C)**, *F* = 5.8, Df = 3, *p* < 0.01 between groups; *F* = 9.6, Df = 2, *p* < 0.0001 for time effect and for interaction, *F* = 2.3, Df = 6, *p* < 0.05. In **(E)**, only time effect was significant with *F* = 88.8, Df = 3, *p* < 0.0001, but there are not significant differences between groups. In **(B,D,F)** one-way ANOVA and Tukey's post test was performed. In **(B)**, statistic values were *F* = 7.4, Df = 3, *p* < 0.001. In **(C)**, *F* = 7.2, Df = 3, *p* < 0.001. In **(F)**, *F* = 6.6, Df = 3, *p* < 0.001, PCS group is significantly different from SM, and from SM INFLIX and PCS INFLIX groups. Values significantly different from controls are indicated by asterisks and from PCS rats by a. ^**^p < 0.01; ^***^p < 0.001; ^a^*p* < 0.05; bb, significant difference between PCS and SM- INFLIX, ^bb^*p* < 0.01.

Spatial learning and memory in the rats were assessed using the Radial maze and the Morris water maze tests. In the radial maze, PCS rats performed more working errors than control rats at day 1 and 2 (Figure 9A). Treatment with infliximab improved performance of PCS rats, which made significantly less (*p* < 0.05) working errors (9 ± 1.5) on day 1 than PCS rats (Figure 9A).

The total number of working errors during the 3 days was higher (*p* < 0.01) in PCS (32 ± 4) than in control rats (16 ± 3). Treatment with infliximab improved performance of PCS rats, which made 23 ± 3 errors, not statistically different from controls (Figure 9B).

PCS rats also made more reference errors (18 ± 1.7) than controls (11 ± 1) in the radial maze at day 1 (Figure 9C). This was also prevented by infliximab. PCS rats treated with infliximab performed significantly less reference errors (14 ± 1, *p* < 0.05) than PCS ras (Figure 9C).

The total number of reference errors during the 3 days was higher (*p* < 0.01) in PCS (45 ± 2) than in control rats (33 ± 3). Treatment with infliximab completely restored performance of PCS rats, which performed 37 ± 2 errors (Figure 9D).

In the Morris water maze, spatial learning was not affected (Figure 9E) but the spatial memory of PCS rats was impaired (Figure 9F). In the memory test PCS remained less time (25 ± 2% of time, *p* < 0.05) in the right quadrant than control rats (36 ± 3% of time). Treatment with infliximab restored spatial memory in PCS rats, which remained 34 ± 2% of time in the right quadrant (Figure 9F).

## Discussion

This study shows that infliximab reduces peripheral inflammation in PCS rats, preventing the increases in pro-inflammatory IL-6, IL-17, and PGE2 and the decrease in anti-inflammatory IL-10. This is in agreement with previous studies in human diseases showing that infliximab reduces peripheral inflammation (Kato et al., 2011; Brunner et al., 2013). It is also shown that reducing peripheral inflammation with infliximab restores spatial learning and memory in rats with HE due to PCS. This suggests that treatment with anti-TNF-a could be a new therapeutic approach to improve cognitive and motor function in patients with HE.

**Figure 10:**
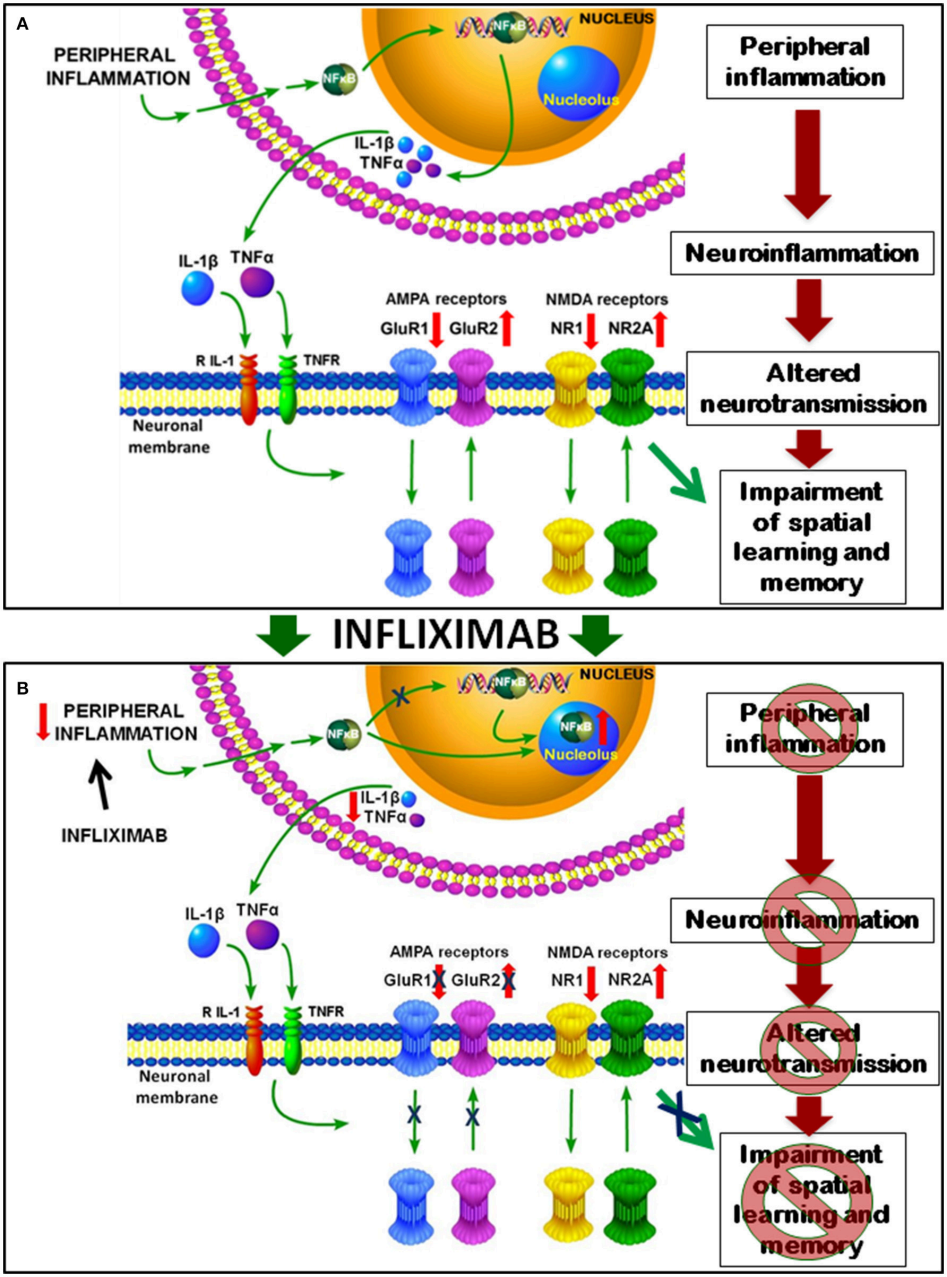
**Proposed model for the mechanisms involved in impairment of spatial learning and memory in rats with HE and for their improvement by infliximab. (A)** Peripheral inflammation in PCS rats results in increased nuclear content of NF-kB in hippocampus which induces the transcription of pro-inflammatory TNF-a and IL-1b, which activate their receptors in neurons of hippocampus. This leads to translocation of AMPA and NMDA receptors. Membrane expression of GluR1 subunit is reduced that of GluR2 increased. For NMDA receptors NR1 is reduced and NR2A increased in membrane in PCS rats. This altered expression of glutamate receptors results in altered neurotransmission which, in turn, leads to impaired spatial learning and memory in the Radial and Morris water mazes. **(B)** Treatment with infliximab reduces peripheral inflammation and p50 subunit of NF-kB content in the nucleus, which is translocated to nucleoli. NF-kB in the nucleoli can't activate transcription of TNF-a and IL-1b, resulting in reduced neuroinflammation in PCS rats treated with infliximab. This reduces activation of TNF-a and IL-1b receptors in the neurons and normalizes membrane expression of AMPA receptors. Normalization of neurotransmission leads to restoration of spatial learning and memory.

We also propose a sequence of events, summarized in Figure 10, by which peripheral inflammation leads to neuroinflammation, altered neurotransmission and cognitive impairment in rats with HE and how treatment with infliximab prevent them.

The present report provides the first demonstration that reducing specifically peripheral inflammation, using anti-TNF-a, which does not cross the blood-brain barrier, prevents the induction of neuroinflammation, the changes in membrane expression of AMPA receptors and associated impairment of spatial learning and memory in rats with HE due to PCS.

The data reported indicate that peripheral inflammation in PCS rats would be the main cause for the induction of neuroinflammation in hippocampus and for impairment of spatial learning.

Peripheral inflammation may induce neuroinflammation by different mechanisms: infiltration of immune cells from the periphery (Gimenez et al., 2006); active transport of some cytokines into the brain parenchyma (Banks et al., 1995) or activation of vagal afferent nerves or direct entry of cytokines at circumventricular regions lacking an intact blood-brain barrier (D'Mello et al., 2009). Blood cytokines may also activate their receptors in endothelial cells and trigger the release of inflammatory factors into the brain (Rummel et al., 2006). For example, in rats injected with LPS, blood IL-6 activates its receptors in endothelial cells leading to activation of STAT3 which increases cyclooxygenase 2 and PGE2 in cerebral cortex (Rummel et al., 2006). We have not found infiltration of blood cells into the brain of PCS rats and have observed that the increase in IL-1b occurs mainly around blood vessels (Figures 5E–G). Moreover, infliximab acts only in the periphery (see Section Introduction) and reduces serum IL-6 in PCS rats. It seems likely that peripheral inflammation in PCS rats would induce neuroinflammation by activation of receptors for pro-inflammatory cytokines in endothelial cells which would transduce the signals to the hippocampus leading to neuroinflammation.

Peripheral inflammation in PCS rats results in reduced IkB levels and increased nuclear content of NF-kB in hippocampus which induces the transcription of pro-inflammatory TNF-a and IL-1b which contribute to impairment of spatial learning (Figure 8A). A similar activation of NF-kB has been observed in ovine hippocampus following LPS-induced peripheral inflammation (Hang et al., 2004; Briscoe et al., 2006) and in cortex of rats with traumatic brain injury, which was also associated with increased TNF-a levels (Hang et al., 2004).

It is noteworthy that the mRNA for TNF-a expression increases in PCS rats mainly in neurons, as shown by *in situ* hybridization. TNF-a and IL-1b are expressed in hippocampal neurons *in vivo* in response to lesions (Tchélingérian et al., 1996) or to pneumococcal meningitis (Izadpanah et al., 2014). *In situ* hybridization studies show that in murine pneumococcal meningitis TNF-a mRNA was first upregulated in astroglial cells but at 18–24 h was strongly increased in hippocampal neurons (Izadpanah et al., 2014). A similar process would occur in hippocampus of rats with HE due to PCS, leading to increased expression of TNF-a in neurons.

The mechanism by which neuroinflammation alters neurotransmission in hippocampus would involve activation of TNF-a and IL-1b receptors in neurons, leading to AMPA and NMDA receptors translocation and altered distribution of its subunits. We show that in PCS rats membrane expression of the GluR1 subunit of AMPA receptors is reduced while that of GluR2 is increased. For NMDA receptors NR1 is reduced and NR2A is increased in membrane in PCS rats. This would be a consequence of increased IL-1b and TNF-a as changes in membrane expression reverse when the content of these pro-inflammatory cytokines are normalized by treatment with infliximab. In support of this, it has been reported that IL-1b reduces membrane expression of the GluR1 subunit of AMPA receptors in hippocampal neurons (Lai et al., 2006). TNF-a also alters membrane expression of AMPA receptors in hippocampus (Ogoshi et al., 2005).

NMDA and AMPA receptors modulate long-term potentiation (LTP) in hippocampus, considered the bases for spatial learning and memory (Morris and Frey, 1997). LTP is impaired in PCS rats (Monfort et al., 2007). The data reported here support that altered membrane expression of AMPA and NMDA receptor subunits would play a main role in impairment of LTP in PCS rats which, in turn, would lead to impaired spatial learning. AMPA receptors activation increases intracellular Na^+^ and, when the GluR2 subunit is lacking also increases Ca^2+^. The presence of the GluR2 subunit prevents entry of Ca^2+^ (Geiger et al., 1995; Liu and Cull-Candy, 2005). In PCS rats, the increase of GluR2 and decrease of GluR1 subunits in the membrane together with the reduced amount of the NR1 subunit of NMDA receptors will reduce Ca^2+^ entry through AMPA receptors, resulting in altered intracellular signaling and neurotransmission, which will finally lead to reduced spatial learning and memory. GluR1 subunit is essential for spatial learning (Sanderson et al., 2008, 2010), thus supporting that the altered membrane expression of GluR1 and GluR2 are main contributors to impaired spatial learning in PCS rats (Figure 10A).

Treatment of PCS rats with infliximab reduces peripheral inflammation and p50 subunit of NF-kB in the nucleoplasm, increasing it in nucleoli. Stark and Dunlop (2005) showed that sub-nuclear compartmentalization regulate NF-kB transcriptional activity in cancer cell lines. Pro-apoptopic treatments translocate the NF-kB p65 subunit to the nucleolus, reducing its levels in the nucleoplasm and decreasing NF-kB transcriptional activity. In contrast, anti-apoptopic treatments such as TNF-a excluded p65 from the nucleolus (Stark and Dunlop, 2005). In this study the effects on NF-kB p50 subunit were not analyzed. In PCS rats p50 is reduced in nucleoli and increased in the nucleoplasm, allowing increased transcription of TNF-a and IL-1b. Treatment of PCS rats with infliximab translocates p50 from nucleoplasm to nucleoli, preventing transcription of TNF-a and IL-1b. This reduces activation of their receptors in the neurons and restores the distribution of AMPA receptors, which return to normal. The normalization of neurotransmission leads subsequently to the improvement in spatial learning and memory (Figure 10B).

In summary, we show that in rats with HE due to PCS, peripheral inflammation leads to neuroinflammation, with increased nuclear NF-kB and expression of TNF-a ad IL-1b in hippocampus, which leads to altered neurotransmission by altering the membrane expression of AMPA and NMDA receptors, which impairs spatial learning and memory. Reducing specifically peripheral inflammation, using anti-TNF-a, which does not cross the blood-brain barrier, reduces neuroinflammation, translocates NF-kB to the nucleoli, normalizes TNF-a and IL-1b in hippocampus, membrane expression of AMPA receptors and spatial learning and memory. These data support that impairment of spatial learning is a consequence of peripheral inflammation and that treatment with anti-TNF-a could be a new therapeutic approach to improve cognitive function in patients with MHE or clinical HE.

## Author contributions

SD treatment of rats and many experiments including PGE-2, western blots and learning tests and obtained part of funding; TB, JF and VH, immunohistochemistry; AC and LT membrane expression; SG, LC, and JG *in situ* hybridization; AA, PCS surgery, learning tests; ML western blots and analysis; VF designed and supervised the work, obtained funding and write the article.

## Funding

Ministerio de Ciencia e Innovación (SAF2011-23051, CSD2008-00005; SAF2014-51851-R), Ministerio de Educacion (FPU13/02492), Generalitat Valenciana (PROMETEO-2009-027, PROMETEOII/2014/033), Danish Council for Independent Research (DFF-1333-00076B), and European Regional Development Funds (ERDF).

### Conflict of interest statement

The authors declare that the research was conducted in the absence of any commercial or financial relationships that could be construed as a potential conflict of interest.
